# A dosimetric comparison of the use of equally spaced beam (ESB), beam angle optimization (BAO), and volumetric modulated arc therapy (VMAT) in head and neck cancers treated by intensity modulated radiotherapy

**DOI:** 10.1002/acm2.12748

**Published:** 2019-10-08

**Authors:** Wan Shun Leung, Vincent W. C. Wu, Clarie Y. W. Liu, Ashley C. K. Cheng

**Affiliations:** ^1^ Department of Health Technology and Informatics The Hong Kong Polytechnic University Kowloon Hong Kong; ^2^ Department of Oncology Princess Margaret Hospital Kowloon Hong Kong

**Keywords:** beam angle optimization, head and neck radiation therapy, IMRT, VMAT

## Abstract

**Introduction:**

Previous studies have shown that the beam arrangement had significant influence on plan quality in intensity modulated radiotherapy (IMRT). This study aimed to evaluate the dosimetric performance of beam arrangement methods by employing equally spaced beams (ESB), beam angle optimization (BAO), and volumetric modulated arc therapy (VMAT) in the planning of five types of head and neck (H&N) cancers treated by IMRT.

**Methods:**

Five plans of different beam arrangement methods were optimized for 119 H&N cancer patients with the prescription of 66–70 Gy for high‐risk planning target volume (PTV), 60 Gy for intermediate risk PTV, 54 Gy for low‐risk PTV using a simultaneously integrated boost method. The five‐beam arrangement methods were: ESB, coplanar BAO (BAOc), noncoplanar BAO (BAOnc), two‐arc VMAT (VMAT2), and three‐arc VMAT (VMAT3). The H&N cancers included cancers of nasopharynx, oral cavity, larynx, maxillary sinus, and parotid. Although the partial arc VMAT could be used in cases where the PTVs were situated at one side of the head such as the parotid, this arrangement was not included because it was intended to include only the beam arrangements that were applicable to all the types of head and neck cancers in the study. The plans were evaluated using a “figure‐of‐merit” known as uncomplicated target conformity index (UTCI). In addition, PTV conformation number and homogeneity index, normal tissue integral dose, and organ at risk (OAR) doses were also used. The mean values of these parameters were compared among the five plans.

**Results:**

All treatment plans met the preset dose requirements for the target volumes and OARs. For nasopharyngeal cancer, VMAT3 and BAOnc demonstrated significantly higher UTCI. For cancer of oral cavity, most beam arrangement showed similar UTCI except ESB, which was relatively lower. For cancer of larynx, there was no significant difference in UTCI among the five‐beam arrangement methods. For cancers of maxillary sinus and parotid gland, the two BAO methods showed marginally higher UTCI among all the five methods.

**Conclusion:**

Individual methods showed dosimetric advantages on certain aspects, and the UTCI of the BAO treatment plans are marginally greater in the case of maxillary sinus and parotid gland. However, if treatment time was included into consideration, VMAT plans would be recommended for cancers of the nasopharynx, oral cavity, and larynx.

## 1 INTRODUCTION

Head and neck cancer is one of the most complicated sites for radiotherapy planning because the planning target volume (PTV) is usually irregular in shape and surrounded by many important organs. Irradiation of the organs at risk (OARs) may cause irreversible side effects such as xerostomia, hearing loss, and trismus that degrade the patient's quality of life.^1^, ^2^, ^3^ Furthermore, head and neck cancers at different locations such as nasopharynx and larynx may lead to different considerations in treatment planning because of their variations in anatomy, body contour, and tissue density combinations.

Intensity modulated radiotherapy (IMRT) has been the main treatment modality for many head and neck cancers due its relatively high target dose conformity and steep dose gradient at target–normal tissue interfaces compared with the conventional three‐dimensional conformal radiotherapy (3DCRT). While the dose distribution in IMRT is largely controlled by the beam modulation using dynamic multileaf collimators (MLC), the beam arrangement including beam number and beam angle employed in the treatment plan have been reported to have significant dosimetric influence in the plan quality in IMRT of many cancers including oesophagus,^4^ lung,^5^ pharynx and larynx,^6^ and nasopharynx.^7^

Equally spaced beam (ESB) arrangement has been commonly used in the early application of IMRT in head and neck cancers after replacing 3DCRT in the early nineties.^8^, ^9^, ^10^ Volumetric modulated arc therapy (VMAT) and beam angle optimization (BAO) in the Eclipse treatment planning system (Varian Medical System, Palo Alto, USA) are the two more recent options in assigning IMRT beams. VMAT is the delivery of IMRT using rotating arc beams,^11^, ^12^ while BAO is the use of a specific optimization algorithm to select the optimum angles of static beams, either coplanar or noncoplanar.

Previous dosimetric studies on the applications of VMAT and ESB in head and neck cancers^13^, ^14^ reported that dual arc VMAT improved the target coverage and OAR sparing in cancers of oropharynx, hypopharynx, and larynx^6^, ^15^ and VMAT produced similar plan quality as ESB arrangement with marked reduction of monitor unit (MU) and shorter treatment delivery time.^13^, ^14^ Studies on BAO are limited. Some of them reported that coplanar BAO arrangement when applied to glioblastoma, prostate, and pancreatic cancers resulted in similar plan quality as ESB arrangement with reduced MU and number of fields.^16^, ^17^ Aside from coplanar beams in IMRT, BAO can also generate noncoplanar beam arrangement. Although noncoplanar IMRT has been reported to reduce the doses to OARs and normal tissues in prostate cancer patients,^18^ its use in the clinical department is uncommon mainly due to the treatment setup inconvenience. However, with the recent emergence of 4Pi radiotherapy with compatible linear accelerators,^19^ it is expected that the use of noncoplanar IMRT will be increased and its potential advantages can be better exercised.

To date, studies on IMRT beam arrangement for head and neck cancers have been limited to specific sites or just any two of the beam arrangement techniques. The optimum beam arrangement, in terms of dosimetric quality, for individual sites of head and neck cancers remains uncertain. Therefore we aimed to conduct a more comprehensive study that evaluated the dosimetric performance of five main IMRT beam arrangement methods on five types of common head and neck cancers that covered the various sites of this body region.

## 2 MATERIALS AND METHODS

A total of 119 adult head and neck cancer patients treated by radical IMRT were randomly selected. They included cancers of the nasopharynx, oral cavity, larynx, parotid gland, and maxillary sinus. Each cancer type consisted of a sample size of 25 except for maxillary sinus, which had 19 due to the limited number of cases available in the clinical department. The distributions of the T and N stages of the patients in each cancer group are summarized in Table 1.

**Table 1:** The distribution of T and N stages of the selected patients.

	Nasopharynx (n = 25)	Oral cavity (n = 25)	Larynx (n = 25)	Maxillary sinus (n = 19)	Parotid (n = 25)
T‐stage					
T1	2	2	0	0	7
T2	3	10	0	0	7
T3	15	11	16	12	8
T4	5	2	9	7	3
N‐stage					
N0	8	3	5	3	10
N1	11	15	17	16	6
N2	6	7	3	0	9

All patients' planning CT data with contoured structures were retrieved from the treatment plan database. Five hypothetical plans, one for each beam arrangement method, were computed for each patient using the Eclipse treatment planning system Version 13.6 (Varian Medical System, Palo Alto, US) by the same dosimetrist. The five‐beam arrangement methods were: equally spaced beams (ESB), coplanar beam angle optimization (BAOc), noncoplanar beam angle optimization (BAOnc), two volumetric modulated arcs (VMAT2), and three volumetric modulated arcs (VMAT3). The ESB arrangement employed nine equally spaced beams at 40° apart as suggested by previous literature.^20^ BAO was performed using Plan Geometry Optimizer (Version 13.6.23) in the Eclipse treatment planning system. BAOc used only coplanar beams while BAOnc included noncoplanar beams. The total number of beams used in both methods ranged between 5 and 9 depending on the beam selection process by the optimization algorithm. VMAT2 consisted of two full arcs, whereas VMAT3 consisted of three full arcs. All treatment plans were planned with 6 MV photon and Millennium MLC. The PTVs were delineated by the oncologist in‐charge. The prescription was 66–70 Gy in 30–35 fractions for high‐risk PTV (PTVH) involving primary tumor or tumor bed and positive nodal involvement. The other two PTVs were intermediate‐risk PTV (PTVI) and low‐risk PTV (PTVL) in the nodal region with prescriptions of 60 and 54 Gy respectively. The PTVs were treated using simultaneously the integrated boost method. The Anisotropic Analytical Algorithm (Version 13.6.23) was used for volume dose calculation and the Photon Optimizer (Version 13.6.23) was used for optimization. For each patient, all the plans were arranged using the same prescribed dose and same set of dose objectives for the target volumes and OARs.

Plans were evaluated by the dose parameters generated from the dose–volume histogram (DVH) of each structure. For the target volumes, the dose parameters were the homogeneity index (HI) and conformation number (CN). The HI was calculated according to the Equation 1 as reported in ICRU 83,^21^ while the calculation of CN as shown in the Equation 2 was adopted from the equation suggested by van't Riet et al.^22^

(1) $$\text{HI}=\left(D_{2\%}-D_{98\%}\right)÷D_{50\%}$$

(2) $$\text{CN}=\frac{V_{T,\;\text{ref}}}{V_{T}}\times \frac{V_{T,\;\text{ref}}}{V_{\text{ref}}},$$

where V_T, ref_ = volume of target receiving a dose equal to or greater than the reference dose, V_T_ = volume of target, V_ref_ = volume receiving a dose equal to or greater than the reference dose.

The OARs considered for dosimetric comparison were the spinal cord, brain stem, and parotid glands (contralateral only for the parotid cancer group) as they were the relatively more critical organs and small changes in dose level would affect the risk of complications. For the spinal cord and brain stem, D_2%_ was used for dose recording, whereas for the parotid gland, D_50%_ and D_mean_ were used. For other OARs such as the optic nerve, cochlea, and pituitary gland, it was expected that they received relatively lower doses; slight differences would not have clinical significance and therefore were not included in this study. The normal tissue dose, which was expressed as the integral dose (in Gy*cm^3^), was calculated by multiplying the D_mean_ of the patient body included while planning CT scan excluding PTVs with the volume of this body region (normal tissue).^23^ In addition, a “Figure of merit”, also known as the uncomplicated target conformity index (UTCI), was used to rank the overall plan quality.^24^ It was calculated by CN × Penalty of organs at risk (P_OAR_) × Penalty of integral dose (P_ID_). The higher the score, the better was the overall plan quality. The CN component of the UTCI was adopted from the Van't Riet study.^22^ The P_OAR_ and P_ID_ were calculated by $e^{\left[-0.05\left(D_{i}-D_{\text{tol}}\right)\right]}$ with D_i_ representing the actual received dose and D_tol_ representing the tolerance dose. Since there was no established tolerance for integral dose, the D_tol_ in the calculation of P_ID_ was taken as the lowest achieved integral dose within the group of the same cancer. The role of P_OAR_ and P_ID_ in the equation was to penalize the UTCI score when the actual OARs of normal tissue dose exceeded their tolerance dose.

Statistical analysis was performed using the SPSS version 20 (IBM Corp, Armonk, NY). All the dose parameters and the UTCI scores were first tested for normality using the Shapiro–Wilk test. The mean values of the dose parameters and UTCI scores for each beam arrangement group were calculated and compared. One‐way repeated measures ANOVA test was used to analyze the differences among the five‐beam arrangement methods. When there was significant difference among them, post hoc Tukey test was applied to further determine the ranking of each method.

## 3 RESULTS

**Figure 1:**
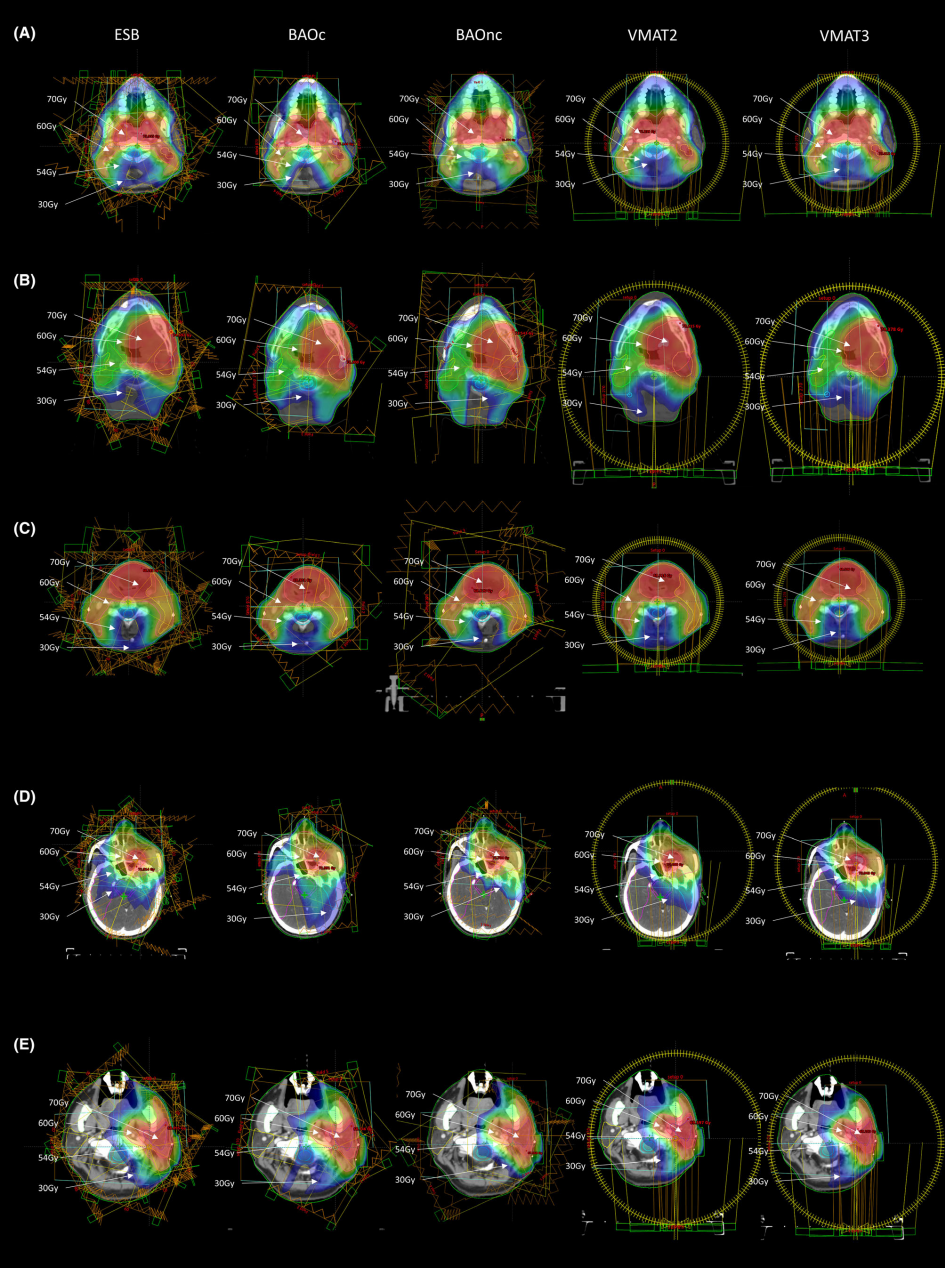
Five types of head and neck (H&N) cancers. Diagram showing the dose distribution of the five types of H&N cancers using different beam arrangement methods. (A) Nasopharynx; (B) Oral cavity; (C) Larynx; (D) Maxilla; (E) Parotid.

All treatment plans met the preset dose requirements for the target volumes and OARs. Examples of dose distribution for the five‐beam arrangement methods for each of the five cancers are shown in Fig. 1.

### 3.1 Cancer of nasopharynx

For PTVH, ESB demonstrated the lowest HI and highest CN (Table 2). ESB and VMAT3 performed relatively better in PTVI, in which ESB showed the highest CN, and VMAT3 showed the lowest HI. For PTVL, VMAT3 showed the lowest HI, whereas both VMAT2 and VMAT3 showed the highest CN. On comparing BAOc and BAOnc, the latter produced higher CN. In addition, VMAT2 and VMAT3 also showed similar target dose distributions. For the OARs and normal tissues, BAO methods delivered relatively lower doses, with BAOnc being the lowest. On the contrary, ESB delivered the highest dose to all OARs and normal tissues. In terms of overall plan quality, BAOnc and VMAT3 demonstrated significantly higher UTCI than the other three beam arrangement methods.

**Table 2:** Comparison of PTVs, OARs dose parameters, and integral dose between IMRT plans of five‐beam arrangements for cancer of nasopharynx (n = 25).

Structure	Dose parameter	ESB (mean ± SD)	BAOc (mean ± SD)	BAOnc (mean ± SD)	VMAT2 (mean ± SD)	VMAT3 (mean ± SD)	Repeated ANOVA *P*‐value	Post hoc test
PTVH	HI	0.06 ± 0.01	0.08 ± 0.01	0.08 ± 0.02	0.06 ± 0.01	0.06 ± 0.01	<0.001	BAOc, BAOnc > ESB, VMAT3, VMAT2
	CN	0.93 ± 0.03	0.82 ± 0.03	0.83 ± 0.03	0.92 ± 0.03	0.91 ± 0.20	<0.001	ESB, VMAT2, VMAT3 > BAOnc, BAOc
PTVI	HI	0.08 ± 0.01	0.07 ± 0.01	0.09 ± 0.01	0.07 ± 0.01	0.06 ± 0.01	<0.001	BAOnc > BAOc, ESB > VMAT2 > VMAT3
	CN	0.90 ± 0.03	0.79 ± 0.04	0.84 ± 0.03	0.89 ± 0.03	0.89 ± 0.03	<0.001	ESB, VMAT2, VMAT3 > BAOnc > BAOc
PTVL	HI	0.07 ± 0.01	0.10 ± 0.01	0.10 ± 0.01	0.06 ± 0.01	0.06 ± 0.01	<0.001	BAOc, BAOnc > ESB > VMAT2, VMAT3
	CN	0.84 ± 0.42	0.83 ± 0.52	0.85 ± 0.41	0.86 ± 0.43	0.86 ± 0.47	0.024	VMAT2, VMAT3, BAOnc, ESB > BAOc
Spinal cord	D_2%_ (Gy)	43.8 ± 1.2	41.9 ± 0.9	41.5 ± 0.8	43.5 ± 1.1	43.7 ± 0.9	<0.001	ESB, VMAT3, VMAT2 > BAOc, BAOnc
Brain stem	D_2%_ (Gy)	51.7 ± 1.8	50.3 ± 1.9	50.2 ± 2.0	51.8 ± 1.8	51.8 ± 2.0	<0.001	VMAT3, VMAT2, ESB > BAOc, BAOnc
Parotid	D_mean_ (Gy)	32.4 ± 5.6	29.9 ± 5.7	29.4 ± 6.1	30.8 ± 5.3	30.7 ± 5.3	<0.001	ESB > VMAT2, VMAT3 > BAOc, BAOnc
	D_50%_ (Gy)	29.9 ± 5.3	28.4 ± 4.8	27.9 ± 5.2	29.1 ± 5.1	28.9 ± 5.1	0.003	
Normal tissue	D_Int_ (×10^4^ Gy cm^3^)	9.5 ± 0.7	7.5 ± 0.7	7.4 ± 0.7	8.4 ± 0.7	8.4 ± 0.7	<0.001	ESB > VMAT2, VMAT3 > BAOc, BAOnc
	UTCI	0.39 ± 0.15	0.41 ± 0.16	0.48 ± 0.20	0.46 ± 0.16	0.49 ± 0.18	<0.001	VMAT3, BAOnc > VMAT2 > BAOc, ESB

BAOc, coplanar beam angle optimization; BAOnc, noncoplanar BAO; CN, conformity number; D_int_, integral dose; ESB, equally spaced beams; HI, homogeneity index; IMRT, intensity modulated radiotherapy; OARs, organ at risks; PTVH, high‐risk PTV; PTVI, intermediate‐risk PTV; PTVL, low‐risk PTV; PTVs, planning target volumes; UTCI, uncomplicated target conformity index; VMAT, volumetric modulated arc therapy; VMAT2, two‐arc VMAT; VMAT3, three‐arc VMAT.

### 3.2 Cancer of oral cavity

For the target volume doses, VMAT3 in general performed better as it achieved lower HI in PTVH and PTVL, highest CN in PTVI and PTVL, and lowest D_2%_ in PTVH and PTVL (Table 3). The rest of the parameters showed relatively small differences and did not reach statistical significance. For the OARs, there was no significant difference in the spinal cord and brain stem doses. For the parotid gland and normal tissues, the two BAO methods (BAOc and BAOnc) gave relatively lower doses and the difference between them was minimal. With regard to UTCI, BAOc, BAOnc, VMAT2, and VMAT3 achieved similar scores, which were higher than that of the ESB.

**Table 3:** Comparison of PTVs, OARs dose parameters, and integral dose between IMRT plans of five‐beam arrangements for cancer of oral cavity (n = 25).

Structure	Dose parameter	ESB (mean ± SD)	BAOc (mean ± SD)	BAOnc (mean ± SD)	VMAT2 (mean ± SD)	VMAT3 (mean ± SD)	Repeated ANOVA *P*‐value	Post hoc test
PTVH	HI	0.07 ± 0.01	0.08 ± 0.02	0.08 ± 0.02	0.06 ± 0.01	0.06 ± 0.01	<0.001	BAOc, BAOnc > ESB, VMAT2, VMAT3,
	CN	0.84 ± 0.04	0.80 ± 0.04	0.80 ± 0.04	0.88 ± 0.03	0.88 ± 0.30	<0.001	VMAT2, VMAT3 > ESB > BAOc, BAOnc
PTVI	HI	0.10 ± 0.02	0.10 ± 0.02	0.10 ± 0.02	0.10 ± 0.02	0.10 ± 0.02	0.378	
	CN	0.84 ± 0.04	0.80 ± 0.04	0.80 ± 0.04	0.86 ± 0.04	0.86 ± 0.04	<0.001	VMAT2, VMAT3 > ESB > BAOc, BAOnc
PTVL	HI	0.10 ± 0.01	0.11 ± 0.01	0.11 ± 0.01	0.10 ± 0.02	0.10 ± 0.02	<0.001	BAOnc, BAOc > ESB, VMAT2, VMAT3
	CN	0.84 ± 0.45	0.81 ± 0.56	0.80 ± 0.44	0.84 ± 0.53	0.84 ± 0.36	<0.001	VMAT3, ESB, VMAT2 > BAOc, BAOnc
Spinal cord	D_2%_ (Gy)	40.3 ± 2.5	39.8 ± 2.8	39.4 ± 2.6	39.1 ± 2.9	39.0 ± 3.1	0.088	
Brain stem	D_2%_ (Gy)	46.2 ± 6.3	46.4 ± 5.2	46.8 ± 5.8	45.7 ± 6.0	45.9 ± 6.2	0.425	
Parotid	D_mean_ (Gy)	28.1 ± 4.7	26.0 ± 5.8	26.0 ± 5.1	28.2 ± 4.6	28.0 ± 4.8	<0.001	VMAT2, ESB, VMAT3 > BAOc, BAOnc
	D_50%_ (Gy)	28.7 ± 4.8	26.9 ± 5.9	27.2 ± 4.6	27.8 ± 4.8	28.0 ± 5.0	0.002	ESB > VMAT3, VMAT2, BAOnc, BAOc
Normal tissue	D_Int_ (×10^4^ Gy cm^3^)	9.7 ± 0.4	8.0 ± 0.6	8.0 ± 0.6	8.7 ± 0.5	8.8 ± 0.5	<0.001	ESB > VMAT2, VMAT3 > BAOc, BAOnc
	UTCI	0.63 ± 0.45	0.77 ± 0.48	0.74 ± 0.48	0.70 ± 0.42	0.70 ± 0.44	0.003	BAOc, BAOnc, VMAT2, VMAT3 > ESB

BAOc, coplanar beam angle optimization; BAOnc, noncoplanar BAO; CN, conformity number; D_int_, integral dose; ESB, equally spaced beams; HI, homogeneity index; IMRT, intensity modulated radiotherapy; OARs, organ at risks; PTVH, high‐risk PTV; PTVI, intermediate‐risk PTV; PTVL, low‐risk PTV; PTVs, planning target volumes; UTCI, uncomplicated target conformity index; VMAT, volumetric modulated arc therapy; VMAT2, two‐arc VMAT; VMAT3, three‐arc VMAT.

### 3.3 Cancer of larynx

There was no significant difference in most of the dose parameters for the target volumes except that VMAT2 and VMAT3 gave a relatively higher CN in the PTVH (Table 4). Similar results were demonstrated in the OAR doses in which no significant difference was observed. For the normal tissues, the two BAO methods showed the lowest dose. However, there was no significant difference in UTCI among the five‐beam arrangement methods.

**Table 4:** Comparison of PTVs, OARs dose parameters, and integral dose between IMRT plans of five‐ beam arrangements for cancer of larynx (n = 25).

Structure	Dose parameter	ESB (mean ± SD)	BAOc (mean ± SD)	BAOnc (mean ± SD)	VMAT2 (mean ± SD)	VMAT3 (mean ± SD)	Repeated ANOVA *P*‐value	Post hoc test
PTVH	HI	0.06 ± 0.01	0.08 ± 0.02	0.07 ± 0.01	0.06 ± 0.03	0.06 ± 0.02	0.100	
	CN	0.88 ± 0.05	0.88 ± 0.04	0.88 ± 0.05	0.90 ± 0.04	0.90 ± 0.40	0.013	VMAT2, VMAT3 > BAOc, ESB, BAOnc
PTVI	HI	0.12 ± 0.02	0.12 ± 0.02	0.13 ± 0.04	0.11 ± 0.03	0.11 ± 0.02	0.040	
	CN	0.82 ± 0.07	0.82 ± 0.08	0.84 ± 0.06	0.86 ± 0.05	0.86 ± 0.05	0.034	
PTVL	HI	0.08 ± 0.03	0.08 ± 0.04	0.08 ± 0.03	0.08 ± 0.03	0.07 ± 0.03	0.482	
	CN	0.84 ± 0.07	0.86 ± 0.05	0.86 ± 0.06	0.84 ± 0.81	0.86 ± 0.06	0.692	
Spinal cord	D_2%_ (Gy)	41.0 ± 2.1	42.2 ± 1.5	42.2 ± 1.8	42.2 ± 1.8	42.3 ± 1.9	0.222	
Brain stem	D_2%_ (Gy)	35.9 ± 19.4	35.1 ± 19.0	35.8 ± 19.5	35.1 ± 19.7	35.1 ± 19.9	0.554	
Parotid	D_mean_ (Gy)	28.5 ± 4.6	27.4 ± 5.1	26.4 ± 4.5	28.2 ± 4.8	27.4 ± 4.2	0.147	
	D_50%_ (Gy)	22.8 ± 4.9	20.3 ± 5.3	20.1 ± 5.5	22.0 ± 6.1	22.2 ± 7.1	0.040	
Normal tissue	D_Int_ (×10^4^ Gy cm^3^)	8.0 ± 0.4	7.3 ± 0.6	7.2 ± 0.6	8.1 ± 0.6	8.3 ± 0.6	<0.001	VMAT3, ESB, VMAT2 > BAOc, BAOnc
	UTCI	1.80 ± 1.61	1.73 ± 1.48	1.99 ± 1.47	1.81 ± 1.87	1.73 ± 1.94	0.527	

BAOc, coplanar beam angle optimization; BAOnc, noncoplanar BAO; CN, conformity number; D_int_, integral dose; ESB, equally spaced beams; HI, homogeneity index; IMRT, intensity modulated radiotherapy; OARs, organ at risks; PTVH, high‐risk PTV; PTVI, intermediate‐risk PTV; PTVL, low‐risk PTV; PTVs, planning target volumes; UTCI, uncomplicated target conformity index; VMAT, volumetric modulated arc therapy; VMAT2, two‐arc VMAT; VMAT3, three‐arc VMAT.

### 3.4 Cancer of maxillary sinus

In general, little difference was observed in PTVs except that the two BAO methods showed relatively higher CN in PTVH and PTVI, and VMAT3 demonstrated the lowest HI for PTVL (Table 5). There was no significant dose difference for the OARs among the five‐beam arrangement methods except for the mean parotid dose, in which the two VMAT plans were relatively higher. For the normal tissues, the two BAO methods demonstrated relatively lower doses. Furthermore, the UTCIs of the two BAO methods were significantly higher than the other three methods.

**Table 5:** Comparison of PTVs, OARs dose parameters, and integral dose between IMRT plans of five‐beam arrangements for cancer of maxilla sinus (n = 19).

Structure	Dose parameter	ESB (mean ± SD)	BAOc (mean ± SD)	BAOnc (mean ± SD)	VMAT2 (mean ± SD)	VMAT3 (mean ± SD)	Repeated ANOVA *P*‐value	Post hoc test
PTVH	HI	0.06 ± 0.01	0.07 ± 0.02	0.07 ± 0.02	0.07 ± 0.02	0.07 ± 0.02	0.206	
	CN	0.87 ± 0.03	0.90 ± 0.02	0.90 ± 0.03	0.87 ± 0.03	0.88 ± 0.03	0.003	BAOc, BAOnc, VMAT3 > VMAT2, ESB
PTVI	HI	0.08 ± 0.01	0.09 ± 0.02	0.09 ± 0.01	0.08 ± 0.03	0.08 ± 0.03	0.286	
	CN	0.79 ± 0.04	0.83 ± 0.06	0.83 ± 0.03	0.81 ± 0.03	0.79 ± 0.03	<0.001	BAOnc, BAOc, VMAT2 > VMAT3, ESB
PTVL	HI	0.09 ± 0.01	0.10 ± 0.01	0.10 ± 0.01	0.09 ± 0.02	0.08 ± 0.02	0.001	BAOnc > BAOc, ESB, VMAT2 > VMAT3
	CN	0.77 ± 0.04	0.79 ± 0.05	0.79 ± 0.03	0.79 ± 0.05	0.78 ± 0.04	0.046	
Spinal cord	D_2%_ (Gy)	41.6 ± 2.2	40.7 ± 2.6	40.9 ± 2.7	41.7 ± 2.5	41.7 ± 2.7	0.044	
Brain stem	D_2%_ (Gy)	51.1 ± 2.1	51.6 ± 1.4	51.6 ± 1.4	50.9 ± 1.7	50.2 ± 3.7	0.266	
Parotid	D_mean_ (Gy)	25.7 ± 5.9	23.9 ± 6.4	23.6 ± 6.7	26.2 ± 6.0	26.3 ± 6.0	<0.001	VMAT3, VMAT2 > ESB, BAOc, BAOnc
	D_50%_ (Gy)	25.7 ± 6.2	25.0 ± 5.7	24.0 ± 6.2	26.6 ± 5.3	26.2 ± 5.7	0.008	
Normal tissue	D_Int_ (×10^4^ Gy cm^3^)	7.5 ± 0.8	6.6 ± 0.8	6.6 ± 0.8	7.3 ± 0.8	7.2 ± 0.8	<0.001	ESB > VMAT2, VMAT3 > BAOnc, BAOc
	UTCI	0.74 ± 0.43	0.99 ± 0.62	1.10 ± 0.72	0.71 ± 0.40	0.71 ± 0.40	<0.001	BAOnc, BAOc > ESB, VMAT2, VMAT3

BAOc, coplanar beam angle optimization; BAOnc, noncoplanar BAO; CN, conformity number; D_int_, integral dose; ESB, equally spaced beams; HI, homogeneity index; IMRT, intensity modulated radiotherapy; OARs, organ at risks; PTVH, high‐risk PTV; PTVI, intermediate‐risk PTV; PTVL, low‐risk PTV; PTVs, planning target volumes; UTCI, uncomplicated target conformity index; VMAT, volumetric modulated arc therapy; VMAT2, two‐arc VMAT; VMAT3, three‐arc VMAT.

### 3.5 Cancer of parotid gland

There was no significant difference among the five‐beam arrangement methods for dose parameters of the PTVs and OARs (Table 6). The two BAO methods delivered the lowest doses to the normal tissues and achieved the highest UTCI.

**Table 6:** Comparison of PTVs, OARs dose parameters, and integral dose between IMRT plans of five‐beam arrangements for cancer of parotid gland (n = 25).

Structure	Dose parameter	ESB (mean ± SD)	BAOc (mean ± SD)	BAOnc (mean ± SD)	VMAT2 (mean ± SD)	VMAT3 (mean ± SD)	Repeated ANOVA *P*‐value	Post hoc test
PTVH	HI	0.06 ± 0.01	0.06 ± 0.01	0.06 ± 0.01	0.07 ± 0.02	0.07 ± 0.01	0.296	
	CN	0.86 ± 0.04	0.88 ± 0.04	0.87 ± 0.4	0.86 ± 0.03	0.86 ± 0.03	0.263	
PTVI	HI	0.07 ± 0.03	0.07 ± 0.02	0.07 ± 0.02	0.09 ± 0.01	0.08 ± 0.02	0.070	
	CN	0.83 ± 0.05	0.87 ± 0.04	0.85 ± 0.03	0.84 ± 0.02	0.82 ± 0.03	0.090	
PTVL	HI	0.09 ± 0.02	0.09 ± 0.03	0.09 ± 0.04	0.10 ± 0.02	0.11 ± 0.03	0.045	
	CN	0.83 ± 0.05	0.84 ± 0.03	0.85 ± 0.04	0.82 ± 0.05	0.84 ± 0.03	0.242	
Spinal cord	D_2%_ (Gy)	34.5 ± 9.4	33.9 ± 8.3	34.2 ± 9.1	34.9 ± 8.9	34.5 ± 9.0	0.319	
Brain stem	D_2%_ (Gy)	35.7 ± 13.4	35.5 ± 12.9	35.5 ± 12.9	36.1 ± 13.2	36.5 ± 11.9	0.390	
Parotid	D_mean_ (Gy)	7.0 ± 3.3	6.5 ± 2.3	6.1 ± 2.4	6.9 ± 3.0	6.8 ± 3.2	0.274	
	D_50%_ (Gy)	8.0 ± 6.2	6.9 ± 4.0	6.8 ± 4.0	7.3 ± 4.4	7.5 ± 4.8	0.051	
Normal tissue	D_Int_ (×10^4^ Gy cm^3^)	8.7 ± 0.7	7.6 ± 1.1	7.7 ± 0.9	8.2 ± 1.1	8.3 ± 0.9	0.002	ESB > VMAT3, VMAT2, BAOnc, BAOc
	UTCI	4.55 ± 1.68	5.66 ± 1.72	5.68 ± 1.81	4.80 ± 1.53	4.88 ± 1.89	<0.001	BAOnc, BAOc, VMAT3, VMAT2 > ESB

BAOc, coplanar beam angle optimization; BAOnc, non‐coplanar BAO; CN, conformity number; D_int_, integral dose; ESB, equally spaced beams; HI, homogeneity index; IMRT, intensity modulated radiotherapy; OARs, organ at risks; PTVH, high‐risk PTV; PTVI, intermediate‐risk PTV; PTVL, low‐risk PTV; PTVs, planning target volumes; UTCI, uncomplicated target conformity index; VMAT, volumetric modulated arc therapy; VMAT2, two‐arc VMAT; VMAT3, three‐ arc VMAT.

## 4 DISCUSSION

Each of the beam arrangement methods had their uniqueness in delivering the tumoricidal dose to the tumor. The equally spaced beam in the ESB method directed the IM beams evenly from all angles around the patient and was considered the best in the treatment of central uniform‐shaped tumors. The VMAT method shared similar characteristics but employed more beams from all angles around the patients and reduced the treatment time.^13^ Because of this, they were expected to deliver higher integral dose to normal tissues.^25^ The VMAT3 had the potential to produce more conformal dose distribution than VMAT2 but required one additional gantry rotation and therefore increased the treatment time. In this study, the BAO methods used 5–9 beams directed from selected angles. Beams that did not have contribution to the plan were eliminated and the beam angles could be tailor‐made for individual patients. As a result, the integral dose and total monitoring units (MU) were lower.^26^ BAOnc had greater freedom to direct the beams to the patient compared with BAOc, but in the expense of longer treatment setup time. Overall, the clinical merit of the current study is that it provides evidence‐based recommendations on the beam arrangement for planners to use in the five types of head and neck cancers.

### 4.1 Cancer of nasopharynx

With regard to the target conformity and homogeneity, ESB performed better in PTVH and PTVI. This could be due to the fact that these target volumes were relatively less irregular in shape than the PTVL and, being more centrally situated at the skull, the evenly distributed intensity modulated IM beams were able to produce relatively more conformal dose distribution. However, for PTVL which extended to both sides of the neck, and was more irregular with an inverted U‐shape (Fig. 1), the BAOnc and VMAT plans demonstrated relatively better dose coverage. The main reason was that with the use of noncoplanar beams in BAO and the greater number of effective beam angles from VMAT, they were both more effective in creating conformal high‐dose volumes covering the irregular target. By the same argument, the OARs were better spared by these two beam arrangement methods.^18^ By combining the performance in the target volumes and OARs, it was logical to see that both BAOnc and VMAT3 achieved relatively better plans among the five‐beam arrangement methods. Overall, VMAT3 would be recommended because it would have a much shorter treatment delivery time compared to BAOnc.

### 4.2 Cancer of oral cavity

In terms of dose coverage to the target volume, VMAT plans in general performed better among the five‐beam arrangement methods as it demonstrated the highest CN in all the PTVs. Since the oral cavity was a relatively large structure, tumors could arise from different locations in the oral cavity ranging from the periphery to the center. The results showed evidence that the VMAT beam arrangement was more flexible to deal with target volume location variation, and its average performance on target coverage was better than the other beam arrangements. Regarding the OARs, the spinal cord and brain stem were located at some distance from the target volume, their doses were relative low, and therefore their differences were small. For the parotid gland, BAO plans provided relatively better sparing and were able to keep the average mean dose below 26 Gy, which was reported to be within the acceptable range of tolerance dose (mean dose 25–30 Gy).^27^ Since the VMAT plans and BAO plans performed better in target volume coverage and parotid gland sparing respectively, it was logical to see their plans achieve similar rank in the UTCI scores. Furthermore, since there was no significant difference between VMAT3 and VMAT2, the addition of extra arc in VMAT3 did not bring any dosimetric advantage and therefore was not necessary. Moreover, BAOc was adequate when compared with BAOnc as including noncoplanar beams did not significantly improve the plan quality. Taking the treatment time into consideration, VMAT2 would be recommended as it shared similar plan quality as the BAOc plans but offered shorter treatment time.

### 4.3 Cancer of larynx

Target volumes in laryngeal cancer were more regular in shape and further away from OARs. All five‐beam arrangement methods performed well on this relatively simple target volume geometry. This was the reason why there was no significant difference in most of the dose parameters of the target volumes, OARs, and UTCI scores. This implied that any one of the beam arrangement methods was effective in treating this cancer. It was worth noting that since the BAO plans restricted the number of beams to below nine, it delivered relatively lower integral dose which might reduce the risk of secondary cancer when compared to the VMAT plans. However, in terms of treatment delivery time, the VMAT plans have the advantage.

### 4.4 Cancer of maxillary sinus

Since tumor of the maxillary sinus was usually located at one side of the head, the evenly distributed beams in ESB and VMAT would irradiate the contralateral structures such as the parotid gland. This phenomenon was reflected in the dosimetric results of the VMAT plans that delivered higher mean parotid gland doses. Besides, the BAO plans which allowed beams mainly directed from the ipsilateral side performed better overall plan quality with the UTCI scores significantly higher than the other three methods. It is logical to consider whether the use of the partial arc VMAT which could also avoid the direct beam entry from the contralateral side could be comparable to the BAO plans. Unfortunately, it was one of the limitations of the current study that the use of the partial arc VMAT was not included, because it was intended to include only the beam arrangements that were applicable to all the types of head and neck cancer in the study. In the current study, VMAT has shown to have comparable results with BAO in the HI and CN of PTV and in the dose to brain stem and spinal cord. Although the advantage of the use of the partial arc VMAT in the cancer of maxillary sinus was not explicitly deduced, the use of partial arc VMAT could possibly achieve the comparable results as in the full arc VMAT while reducing the disadvantages attributable to the beam entry from the contralateral side. Therefore, the current results were not against the use of partial arc VMAT in the cancer of maxillary sinus.

### 4.5 Cancer of parotid gland

Target volumes of the parotid gland tumor were usually followed a triangular shape and would not poses great difficulty to the various beam arrangements. This was reflected in the dosimetric results of the target volumes in which there was no significant difference in all the dosimetric parameters among the five‐beam arrangement methods. Similar to the maxillary sinus cancer, parotid tumors are situated at the lateral aspect of the head, and this would be a disadvantage for the ESB and VMAT beam arrangement. Relatively higher doses were found in the contralateral parotid gland in these plans although the differences did not reach statistical significance. Similar to the explanation for the maxillary sinus cancer, the integral dose in the BAO plans were significantly lower, and this led to overall better UTCI scores in these two plans. This echoed the study by Yirmibesoglu et al^28^ who reported that four‐field ipsilateral IMRT techniques provided excellent coverage while maximally sparing the contralateral parotid gland and submandibular gland. As a result, BAOc plans would be recommended as it achieved the same plan quality as the BAOnc but offered simpler treatment setup procedure. Furthermore, by the same argument as stated for the cancer of maxillary sinus, the results of the present study were not against the use of partial arc VMAT in the cancer of parotid gland.

## 5 CONCLUSION

The five‐beam arrangement methods produced acceptable plans for all the five groups of head and neck cancer patients. Partial arc VMAT was not included in the beam arrangement methods because it was not commonly applied in centrally located cancers, therefore it could not be used for comparison among the five groups of head and neck cancers. The results showed that individual methods produced dosimetric advantages on certain aspects, and the UTCL scores were marginally greater in the BAO method in the cancers of the maxillary sinus and the parotid gland. However, if the treatment time was included into consideration, VMAT plans would be recommended for cancers of nasopharynx (VMAT3), oral cavity, and larynx (VMAT2).

## CONFLICT OF INTEREST

The authors declare no conflicts of interest with respect to the content of this manuscript.
